# New Perspective on the Geographic Distribution and Evolution of Lymphocytic Choriomeningitis Virus, Central Europe

**DOI:** 10.3201/eid2710.210224

**Published:** 2021-10

**Authors:** Alena Fornůsková, Zuzana Hiadlovská, Miloš Macholán, Jaroslav Piálek, Joëlle Goüy de Bellocq

**Affiliations:** Institute of Vertebrate Biology of the Czech Academy of Sciences, Brno, Czech Republic (A. Fornůsková, J. Piálek, J. Goüy de Bellocq);; Institute of Animal Physiology and Genetics of the Czech Academy of Sciences (Z. Hiadlovská, M. Macholán);; Czech University of Life Sciences Prague, Prague (J. Goüy de Bellocq)

**Keywords:** viruses, lymphocytic choriomeningitis virus, central Europe, *Mus musculus musculus*, *Mus musculus domesticus*, zoonoses, Czech Republic, Germany

## Abstract

Lymphocytic choriomeningitis virus (LCMV) is an Old World mammarenavirus found worldwide because of its association with the house mouse. When LCMV spills over to immunocompetent humans, the virus can cause aseptic meningitis; in immunocompromised persons, systemic infection and death can occur. Central Europe is a strategic location for the study of LCMV evolutionary history and host specificity because of the presence of a hybrid zone (genetic barrier) between 2 house mouse subspecies, *Mus musculus musculus* and *M. musculus domesticus*. We report LCMV prevalence in natural mouse populations from a Czech Republic–Germany transect and genomic characterization of 2 new LCMV variants from the Czech Republic. We demonstrate that the main division in the LCMV phylogenetic tree corresponds to mouse host subspecies and, when the virus is found in human hosts, the mouse subspecies found at the spillover location. Therefore, LCMV strains infecting humans can be predicted by the genetic structure of house mice.

Lymphocytic choriomeningitis virus (LCMV) is the prototype of the family Arenaviridae. Its genus *Mammarenavirus* is associated with rodent-transmitted diseases in humans, including agents of hemorrhagic fevers, such as Lassa virus and Junin virus (*1*). In immunocompetent persons, LCMV infection is typically asymptomatic but can cause nonspecific febrile illness or aseptic meningitis. However, LCMV infection can cause severe congenital disease, and it has been reported at the origin of 6 clusters of severe or fatal disease among solid organ recipients in the past 20 years (*2*).

All mammarenaviruses are enveloped ambisense RNA viruses. Their genome (≈11-kb) is composed of 2 segments, each encoding 2 proteins in nonoverlapping open reading frames (ORFs); the large 7.2-kb segment encodes the Z matrix and the large polymerase proteins, and the small 3.4-kb segment encodes the glycoprotein and nucleoprotein (*1*).

The primary host reservoirs of LCMV are house mice (*Mus musculus*), although the virus has been reported in other rodents, and experimental infections have been described in other mammals, such as rabbits, dogs, and pigs (*3*–*5*). The house mouse is a complex of several subspecies. The most widespread subspecies are *M. musculus musculus*, found from central and northern Europe to the Far East; *M. musculus domesticus*, which is found in western and southern Europe, northern Africa, the Middle East, and, more recently, in North and South America, southern Africa, Australia, and Oceania because of passive transport with humans; and *M. musculus castaneus,* which is found in central and southeastern Asia (*6*,*7*) (Figure 1). These subspecies are not reproductively isolated, and several regions of secondary contact hybridization exist, including a >2,500 km–long region in Europe extending from Scandinavia to the Black Sea in which *M. musculus musculus* and *M. musculus domesticus* mice have contact (Figure 1). In this region, the 2 subspecies form a hybrid zone with a barrier to gene flow between them (*6*,*9*,*10*). Recent studies have shown that such hybrid zones can also act as barriers for the organisms’ pathogens (*11*–*14*). Hybrid zones are thus useful natural settings to study the limit of host specificity, which is pivotal to understanding the geographic distribution of pathogens and their potential for spillover. For example, 2 mammarenaviruses, Morogoro virus and Gairo virus, are each confined to 1 of the 2 subtaxa of their host, the Natal mulimammate mouse (*Mastomys natalensis*), even though the host hybridizes in Tanzania (*13*,*15*).

**Figure 1:**
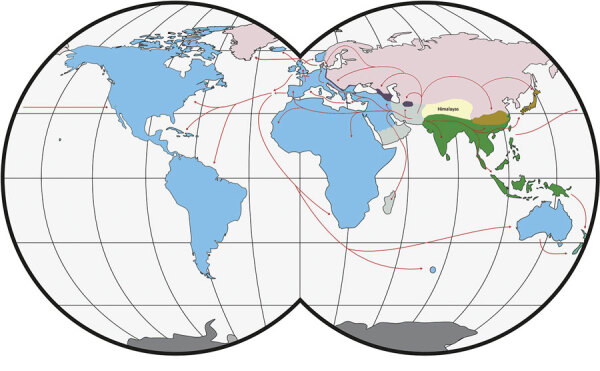
Worldwide distribution of *Mus musculus* mouse subspecies. Colors indicate subspecies ranges: green and tan, *M. musculus castaneus*; blue and purple, *M. musculus domesticus*; pink, *M. musculus musculus* ; gray, central populations and *M. musculus gentilulus*. Note that house mice may not be found throughout the complete extent of some areas (e.g., subarctic regions, the Sahara Desert, and the Amazon rainforest). The purple, and gray areas indicate regions of hybridization. Red arrows indicate inferred routes of historical migrations and recent movements in association with humans. Adapted from (*7*,*8*). Copyright ©2012 Springer-Verlag. All rights reserved. Adapted with permission from Springer Science and Business Media and Michael Nachman.

In 2010, Albariño et al. (*16*) investigated the genetic diversity and distribution of LCMV variants by analyzing 29 genomes. They demonstrated that LCMV is highly diverse and forms 4 distinct lineages (I–IV) but found little correlation of those lineages with time or place of isolation. From their dataset, only 3 strains (Marseille12-2004, Yale-1977, and Michigan-2005) originated from wild mice, but those strains were not assigned to subspecies. Furthermore, the place of isolation is a poor proxy for the origin of spillover to human hosts. For example, focusing on lineage II of Albariño et al., strains M1 and M2 were isolated in Japan in 2005, but came from a wild-derived strain originating from *M. musculus musculus* mice caught in Illmitz, Austria, in 1985 (*17*). Likewise, the Dandenong-Yugoslavia LCMV strain (*18*) was isolated in Australia from a human spillover, but that person returned from the former Yugoslavia before becoming ill and dying. The Bulgaria 1956 strain (*19*) was isolated from a human spillover, but geographic origin was not mentioned in the original study; a contact of that patient was treated for the same symptoms in a hospital in Vidin, Bulgaria, suggesting spillover origin in northwestern Bulgaria. Finally, the last LCMV strain in lineage II, LE-FRANCE (*20*), was isolated from a pregnant woman in France (i.e., within *M. musculus domesticus* mouse territory), but the person worked in a pet store, making strain origin uncertain because other rodent species, especially hamsters, are known to be LCMV carriers (*3*,*4*,*20*). In summary, for 3 of 4 LCMV strains in lineage II, the potential spillover origin is consistent with *M. musculus musculus* mouse territory despite diverse viral isolation locations. Similarly, in LCMV lineage I, strains were found in laboratory mice, essentially of *M. musculus domesticus* origin (*21*); wild mice; or in primate (including human) spillovers in the United States or western Europe, and were thus consistent with *M. musculus domesticus* mouse origin (*22*). LCMV lineage IV consists only of strains isolated from woodmice (*Apodemus sylvaticus*) from Spain. Given these observations, we hypothesized that host specificity could be a better predictor of LCMV genetics than the place or time of LCMV strain isolation.

In this study, we test the hypothesis that LCMV phylogenetic clustering reflects specificity to its host reservoirs by investigating the diversity of LCMV in central Europe across the house mouse hybrid zone (HMHZ). We also update the phylogenetic analysis of LCMV from Albariño et al. (*16*) by complementing their dataset with LCMV genomes sequenced in the last decade and with our data.

## Material and Methods

### Sampling and Mouse Genotyping

**Figure 2:**
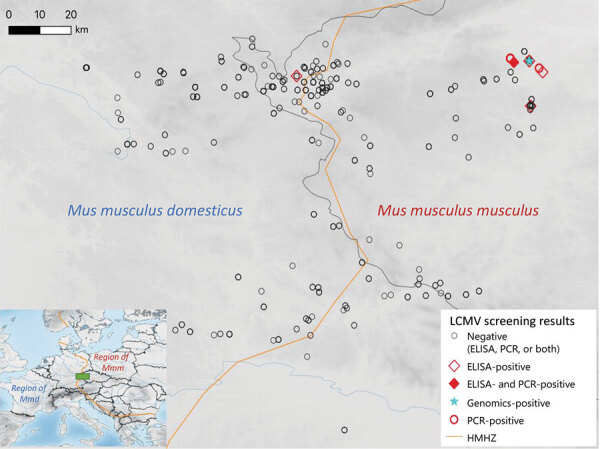
Tested localities for LCMV, Central Europe. The center of the HMHZ is represented by the orange line. The green rectangle n the inset map shows the sampling area, located in the center of the HMHZ. HMHZ, house mouse hybrid zone; LCMV, lymphocytic choriomeningitis virus.

A total of 748 house mice (410 *M. musculus domesticus* and 338 *M. musculus musculus*) from 179 localities (100 for *M. musculus domesticus* and 79 for *M. musculus musculus*) were trapped in farms during 2008–2019 across a 145-km by 110-km belt stretching from northeastern Bavaria (Germany) to western Bohemia (Czech Republic), a region in which these mouse subspecies meet and form the HMHZ (*23*) (Figure 2; Appendix 1 Table 1). Tissue samples were preserved in liquid nitrogen and later stored at −80°C as described in Goüy de Bellocq et al. (*24*). Mice were identified on the basis of a set of diagnostic markers as in Macholán et al. (*23*) or on the basis of 1,401 single-nucleotide polymorphism (SNP) markers (*25*) or 0.62 million SNP markers (*26*) (Appendix 1 Table 1). Each individual mouse’s hybrid index (HI) was estimated as the proportion of *M. musculus musculus* alleles. We considered all mice with HIs <0.5 as *M. musculus domesticus*–like and those with HIs >0.5 as *M. musculus musculus*–like.

### LCMV Serologic and Molecular Screening

We screened 291 blood plasma samples collected from 100 localities during 2008–2011 for LCMV antibodies by using the ELISA kit IM-698 C-EB (XpressBio, https://xpressbio.com). We used 100 μL of 1:50 diluted serum for the reaction according to the manufacturer’s instructions. In addition, we extracted RNA from 616 spleen or salivary gland samples by using RNeasy Mini kit (QIAGEN, https://www.qiagen.com). We reverse-transcribed the RNA samples collected in 2008–2013 by using the Applied Biosystems High-Capacity RNA-to-cDNA Kit (ThermoFisher Scientific, https://www.thermofisher.com) in 10 μL final volume. We screened for LCMV by targeting a 340-nt fragment of the large gene by using primers from Vieth et al. (*27*), because these primers detected LCMV in a previous study (*28*). Samples were screened with the Multiplex PCR kit (QIAGEN) in a final volume of 15 μL by using 2 μL of cDNA and following the manufacturer’s instructions. To increase assay sensitivity, we also designed primers for a nested PCR assay on the basis of LCMV sequences available in GenBank and targeting 442 nt in a part of the large gene partially overlapping with the region described previously. We tested 96 samples with both assays and results showed the same number of positive samples. However, the first assay (i.e., Vieth et al. primers) showed higher sensitivity (stronger band in 1.5% agarose gels); therefore, we selected that assay to screen the complete dataset. However, we used the second assay for Sanger sequencing of all positive samples to obtain longer final large fragment (659–665 nt resulting from merging both assay outputs). We screened the 2019 RNA samples with Vieth et al. primers by using Invitrogen SuperScript IV One-Step RT-PCR System (ThermoFisher Scientific) in a final volume of 20 μL and using 3 μL of extracted RNA. We attempted additional amplifications in positive samples to sequence parts of the glycoprotein and nucleoprotein genes (Appendix 2 Table). We purified PCR products and Sanger sequenced in both directions by using Eurofins Genomics (https://eurofinsgenomics.com).

### Whole-Genome Sequencing and Assembly of LCMV Viruses

We selected 2 positive samples from localities 10 km apart: sample SK1042 from Kryry, Czech Republic (KRY1) and sample SK1194 from Nepomyšl, Czech Republic (NEPO1), for whole-genome sequencing. We extracted RNA from lung and liver specimens by using the viral enrichment protocol described in Goüy de Bellocq et al. (*29*). The cDNA synthesis, library preparation, and sequencing (BGI Genomics, https://www.bgi.com) were carried out as described in Goüy de Bellocq et al. (*30*). After read demultiplexing, quality filtering, and trimming, 48,209,592 paired-end reads were available for SK1042, and 39,228,040 paired-end reads were available for SK1194. We used only 10,000,000 paired-end reads for a de novo assembly by iterative mapping with Geneious Mapper in Geneious 11 (Geneious, https://www.geneious.com). We enriched for LCMV reads in silico by removing all reads that mapped to mouse reference genome GRCm38. The LCMV iterative mapping was seeded with the 340 nt of the large gene obtained by Sanger sequencing and a 74-nt sequence conserved among LCMV strains for the Z gene. For the small segment, we generated 2 small seed reference sequences of ≈150 nt in the glycoprotein and nucleoprotein by first mapping the paired-end reads to LCMV strain Traub (from *M. musculus domesticus* mice). We confirmed the sequence of the intergenic region of the large segment by Sanger sequencing designing primers in the neighboring coding regions (Appendix 2 Table). After assembly, we ensured the seeding had not influenced the output. Finally, as part of a viral metagenomic study of digestive tract samples taken from mice in the HMHZ (J. Goüy de Belloq, unpub. data), we detected 229-nt and 458-nt contigs that matched via BLAST (https://blast.ncbi.nlm.nih.gov/Blast.cgi) with the large and glycoprotein gene of LCMV and Dandenong virus in a pooled sample of 3 mice coming from Buškovice (BUS2) collected in 2014. We included these 2 sequences in the current study.

### Phylogenetic Analyses

LCMV nucleotide sequences were aligned with the sequence coding parts of the nucleoprotein, glycoprotein, and large genes of other strains available in GenBank (Appendix 1 Table 2). We included all strains analyzed in Albariño et al. (*16*), augmented with 11 recently published genome sequences that included LCMV variants of *M. musculus domesticus* mouse origin from France, Gabon, and French Guiana (*28*,*31*,*32*). Nucleotide alignment was based on amino acid sequences in Geneious Prime 2019.2 (Geneious) by using the ClustalW algorithm. We used the Bayesian Information Criterion in MEGA X (*33*) to evaluate models of nucleotide and amino acid substitution. The best-fit model was general time reversible plus invariate sites plus gamma distribution for all 3 genes on the basis of the nucleic acid dataset, Jones-Taylor-Thornton plus invariate sites plus gamma for large and nucleoprotein genes, and Le Gascuel plus gamma for the glycoprotein gene for the amino acid dataset. We performed phylogenetic analyses on nucleic acid and amino acid sequences by using Bayesian inference using MrBayes version 3.2.7 (*34*). For the amino acid sequence dataset, we only included LCMV sequences in which a large proportion of the coding genes were sequenced. We conducted default priors for all parameters and 2 independent runs with 10 million generations per run and sampled trees and parameters every 500 generations, discarding the first 25% of sampled trees as burn-in. We used Bayesian posterior probabilities (PP) to assess node support and the complete genome of Lunk virus from African *Mus minutoides* as an outgroup. We prepared tree figures in FigTree version 1.4.4 (http://tree.bio.ed.ac.uk/software/figtree).

## Results

### Rodent Sampling and LCMV Detection

We found 7 positive samples among the 291 samples (160 *M. musculus domesticus* and 131 *M. musculus musculus*) tested with ELISA for a prevalence of 2.4%. A total of 6 positives were revealed in *M. musculus musculus* mouse territory: 3 in Buškovice (BUS2, 2008), 1 in Nepomyšl (NEPO1, 2009), 1 in Kryry (KRY1, 2009), and 1 in Žihle (2010). All these specimens had HI >0.96, indicating almost pure *M. musculus musculus*. The single positive specimen from *M. musculus domesticus* territory mouse (HI = 0.20) was captured in locality Starý Rybník Vepřín (SRYV) (2009), 3.7 km from the center of the HMHZ. By using the molecular LCMV screening, we found 5 positive samples out of 616 analyzed (prevalence = 0.8%) (Table). All were from *M. musculus musculus* mouse territory with HI >0.97: 4 specimens from NEPO1 (2008 and 2009) and 1 specimen from KRY1 (2009). One specimen from NEPO1 (SK1042; HI = 0.98) was found positive by both serologic and molecular screening approaches. All the positive localities confirmed genetically were located within a 12-km^2^ area. The detection of RNA-positive samples in 2009 and 2010 in NEPO1 and the repeated finding of positive specimens in BUS2 by ELISA in 2008 and 2014 (J. Goüy de Belloq, unpub. data) suggest that LCMV is locally endemic in *M. musculus musculus* mouse territory, persisting within farms over several years.

**Table:** Overview of sampled localities and tested mice in study of geographic distribution and evolution of lymphocytic choriomeningitis virus, central Europe

Subspecies	No. sampled localities	No. tested by PCR/no. positive	No. tested by ELISA/no. positive	No. genomic sequences	Total positive samples
*Mus musculus domesticus*	100	335/0	160/1	0	1
*M. musculus musculus*	79	281/5	131/6	3	11
Total	179	616/5	291/7	3	12

### Characterization of the Full Genomes of LCMV from the Czech Republic

We obtained LCMV whole-genome sequences from 2 mice samples. Because the partial large sequences of the 4 samples from NEPO1 were identical, we characterized the genome of only 1 LCMV sample (SK1194). The other sample (SK1042) was from the locality KRY1, 10 km from NEPO1. At the 3′ end of the large segment of the variant SK1194, ≈19 noncoding nucleotides were missing. Each of the 2 segments showed the 2 ORFs typical for mammarenavirus separated by typical stem-loop structures. The complete large segment was 7232 nt (for strain SK1042) and contained 2 ORFs: the Z ORF (270 nt) encodes a 90-aa zinc-finger protein, whereas the large ORF (6627 nt) encodes a 2209-aa RNA-dependent RNA polymerase. The complete small segment was 3380 nt for SK1194 and 3381 nt for SK1042 and contained 2 ORFs: the glycoprotein ORF (1494 nt) coding for a 498-aa glycoprotein precursor and the nucleoprotein ORF (1674 nt) encoding a 558-aa nucleoprotein. The pairwise nucleotide divergence between the 2 variants was 8.4% for the small segment and 9.6% for the large segment.

### Phylogenetic Analysis

**Figure 3:**
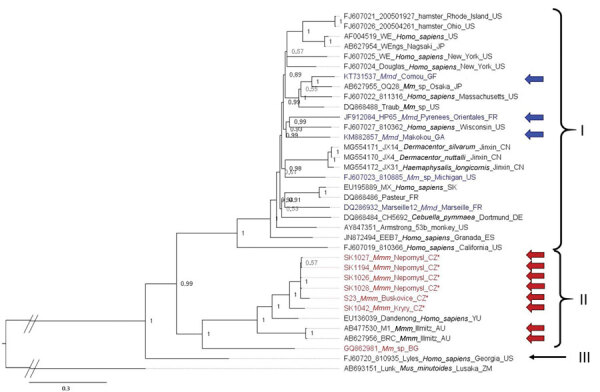
Phylogenetic analysis performed on nucleic acid sequences of large gene of lymphocytic choriomeningitis virus (LCMV) sequences using Bayesian inference. Bayesian posterior probabilities were used to assess node support. Lunk virus from *Mus minutoides* (Africa) was used as outgroup. All sequences obtained in this study were submitted to GenBank (accession nos. MZ568450–7, MZ558311–3, MZ568449). Names of LCMV strains are composed of GenBank accession number, strain name, host species, and place and country of origin (if known) or isolation. Country code is defined as ISO code (https://countrycode.org). Colors indicate LCMV strains isolated from wild rodents where there is a match between expected mouse subspecies on the basis of geographic region and sampling area: blue, *Mus musculus domesticus*; red, *M. musculus musculus*. Arrows indicate known origin of mice subspecies on the basis of genetic data, asterisks (*) indicate LCMV strains from this study, and lineages are indicated by roman numerals. Scale bar indicates nucleotide substitutions per site. Mmd, *M. musculus domesticus*; Mmm, *M. musculus musculus*; Mmm_lab, laboratory mouse strain derived from *M. musculus musculus*; Mm_lab, laboratory mouse strain; Mm_sp, *Mus musculus* spp.

We analyzed the large, glycoprotein, and nucleoprotein genes separately and highlighted the position of the new LCMV variants found in *M. musculus musculus* mice from the Czech Republic and of the variants known to have been isolated from wild *M. musculus domesticus* mice. For the large nucleotide tree (Figure 3), the topology of the phylogeny is similar to that of Albariño et al. (*16*); 2 lineages are highly supported (PP = 0.99): I (harboring 24 LCMV strains) and II (10 LCMV strains). Lineage III, which consisted of a single sequence isolated from a human in Georgia (USA), has a highly supported basal position (PP = 1). All sequences from Czech Republic *M. musculus musculus* mice form a sister clade to Dandenong virus within lineage II, the lineage we hypothesized to originate from *M. musculus musculus* mice. The 3 variants from *M. musculus domesticus* mice are found scattered within the highly supported lineage I (PP = 0.99). The internal topology of lineage I is not resolved and consists of sequences originating from various hosts and regions. Among these clades, a subset was isolated from primate spillovers, likely from local mice in the United States, Spain, France, and Germany (i.e., within *M. musculus domesticus* mouse territory). The other strains that do not overlap with the range of *M. musculus domesticus* mice have either been isolated from cell culture (strains from Japan or Slovakia) or ticks (strains from China). In the phylogenetic tree constructed on the basis of large amino acid sequences, the position of lineage III is again well supported but is basal to lineages I and II, both with highly supported monophyly (PP = 1) (Appendix 2 Figure, panel A).

**Figure 4:**
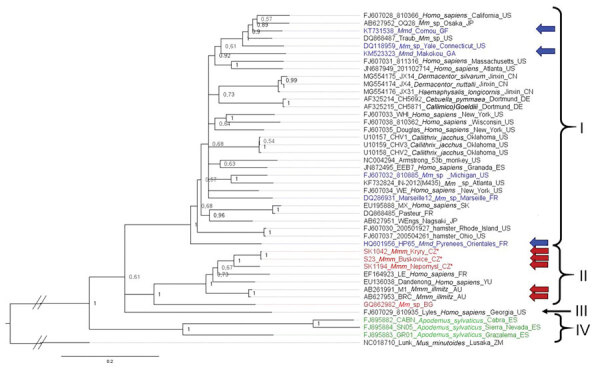
Phylogenetic analysis performed on nucleic acid sequences of glycoprotein gene of lymphocytic choriomeningitis virus (LCMV) sequences using Bayesian inference. Bayesian posterior probabilities were used to assess node support. Lunk virus from *Mus minutoides* (Africa) was used as outgroup. All sequences obtained in this study were submitted to GenBank (accession nos. MZ568450–7, MZ558311–3, MZ568449). Names of LCMV strains are composed of GenBank accession number, strain name, host species, and place and country of origin (if known) or isolation. Country code is defined as ISO code (https://countrycode.org). Colors indicate LCMV strains isolated from wild rodents where there is a match between expected mouse subspecies on the basis of geographic region and sampling area: blue, *Mus musculus domesticus*; red, *M. musculus musculus*. Arrows indicate known origin of mouse subspecies on the basis of genetic data, asterisks (*) indicates LCMV strains from this study, and lineages are indicated by roman numerals. LCMV strains isolated from *Apodemus sylvaticus* are indicated in green (lineage IV). Scale bar indicates nucleotide substitutions per site. Mmd, *M. musculus domesticus*; Mmm, *M. musculus musculus*; Mmm_lab, laboratory mouse strain derived from *M. musculus musculus*; Mm_lab, laboratory mouse strain; Mm_sp, *Mus musculus* spp.

**Figure 5:**
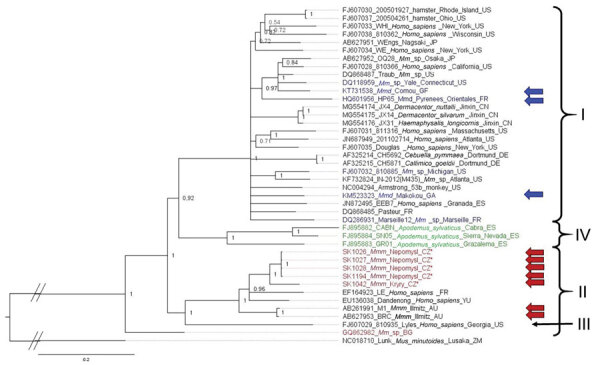
Phylogenetic analysis performed on nucleic acid sequences of nucleoprotein gene of lymphocytic choriomeningitis virus (LCMV) sequences using Bayesian inference. Bayesian posterior probabilities were used to assess node support. Lunk virus from *Mus minutoides* (Africa) was used as outgroup. All sequences obtained in this study were submitted to GenBank (accession numbers: MZ568450–7, MZ558311–3, MZ568449). Names of LCMV strains are composed of GenBank number, strain name, host species, and place and country of origin (if known) or isolation. Country code is defined as ISO code (https://countrycode.org). Colors indicate LCMV strains isolated from wild rodents where there is a match between expected mouse subspecies on the basis of geographic region and sampling area: blue, *Mus musculus domesticus*; red, *M. musculus musculus*. Arrows indicate known origin of mice subspecies on the basis of genetic data, asterix indicates LCMV strains from this study, and lineages are indicated by roman numerals. LCMV strains isolated from *Apodemus sylvaticus* are indicated in green (lineage IV). Scale bar indicates nucleotide substitutions per site. Mmd, *M. musculus domesticus*; Mmm, *M. musculus musculus*; Mmm_lab, laboratory mouse strain derived from *M. musculus musculus*; Mm_lab, laboratory mouse strain; Mm_sp, *Mus musculus* spp.

The phylogenetic position of the sequences from Czech Republic *M. musculus musculus* and wild *M. musculus domesticus* mice in our nucleoprotein and glycoprotein gene trees corresponds to that in the large gene tree. An additional clade, clade IV, is composed of strains isolated from the woodmouse (*Apodemus sylvaticus*). All 4 glycoprotein lineages based on amino acid sequences were highly supported (PP = 1) (Appendix 2 Figure, panel B), whereas the phylogenetic signal at the nucleotide level seems to be compromised by homoplasy, resulting in trichotomy between lineages I, II, and III (Figure 4). A similar pattern can be seen in the phylogenetic trees based on the nucleoprotein gene but with low support. Phylogenetic relationships between lineages are not resolved, demonstrating differences with regard to the type of data. The basal position of lineage IV (woodmouse) to other lineages is well supported (PP = 1) on the basis of amino acid sequences (Appendix 2 Figure, panel C). By contrast, nucleotide sequences show lineage IV as sister group to lineage I (PP = 0.92) and lineage III clustering with lineage II (PP = 1), whereas the strain from Bulgaria is basal to all other ingroup lineages (PP = 1), suggesting that homoplasy at the nucleic acid level affects the phylogenetic signal (Figure 5).

## Discussion

We found LCMV at low prevalence in wild mice in central Europe, and all genetically confirmed cases clustered within a small geographic region in the *M. musculus musculus* mouse side of the HMHZ. This low prevalence prevents direct inference of the zone as a barrier to LCMV exchange between the mouse subspecies in nature. However, our phylogenetic analyses, which included new LCMV variants from the Czech Republic, 3 variants sequenced from wild *M. musculus domesticus* mice, other LCMV variants sequenced during the last decade, and supplemented with published data, support the hypothesis that LCMV lineage I harbors viruses originating from *M. musculus domesticus* mice and lineage II includes viruses primarily found in *M. musculus musculus* mice.

The low prevalence of LCMV observed in central Europe is not uncommon. In wild mice, this prevalence has been shown to be variable, ranging from 0 to 25% (*2*), but most studies have reported low prevalence and patchy distribution. For example, Ackermann et al. (*34*) found an overall prevalence of 3% in wild mice from Germany, with 65 LCMV-positive specimens from 44 localities, but despite extensive sampling efforts in Bavaria as a whole (380 mouse samples over 70,000 km^2^), no LCMV-positive mice were found there (*35*). We also failed to detect any positive LCMV samples in Bavaria (*M. musculus domesticus* mouse region). The low prevalence of LCMV is comparable to other mammarenaviruses (e.g., Gairo virus and Morogoro virus in *Mastomys natalensis* mice in Tanzania) (*13*,*15*).

We reported LCMV infection in Buškovice in 2008 and 2014; however, we were unable to demonstrate genetic turnover during that period. Commensal mouse populations are usually structured to local subpopulations or demes, with a dispersal scale of ≈1 km^2^ (*36*,*37*). Because LCMV can spread both horizontally and vertically, maintenance of the virus within a deme over several years seems plausible. Whether LCMV variants are still present in the 12 km^2^ area is not certain. If so, targeted rodent control measures could feasibly decrease or eliminate LCMV risk for humans in this geographic area.

Albariño et al. (*16*) described 4 main LCMV lineages. Our results suggest that >3 of these lineages correspond to different host subspecies: lineage I to *M. musculus domesticus*, lineage II to *M. musculus musculus*, and lineage IV to *Apodemus sylvaticus*. We make no claim regarding the origin of lineage III, a single isolate from a human in Georgia (USA) (i.e., theoretically *M. musculus domesticus* mouse territory). We suggest more highly divergent lineages are likely to be discovered corresponding to rodent species, subspecies, and cryptic taxa. A new LCMV strain was recently reported from human serum in southern Iraq (*38*), but its phylogenetic position cannot be resolved; only a short fragment of the large gene (395 nt) is available in GenBank. This new LCMV strain is likely to cluster in clade I because *M. musculus domesticus* is the expected house mouse subspecies in southern Iraq (*39*–*41*). Uncertainty persists with respect to 4 LCMV strains clustered within lineage I of expected *M. musculus domesticus* mouse origin; JX14, JX4, and JX31 were isolated from ticks in 2015 from a coastal area in Jinxin, Jilin Province, northeastern China, and strain OQ28 was sequenced in 1990 from a wild mouse (*M. musculus*) captured in Osaka, Japan (*42*,*43*). In both regions, mice of subspecies other than *M. musculus domesticus* were reported. *M. musculus musculus* mice occur in northern China (*44*), whereas in Japan, mice are generally identified as *M*. *musculus castaneus* or *M. musculus* *molossinus* (*45*). However, the *M. musculus domesticus* mouse is known to be a successful invasive species because of ancient and recent human mobility, and its introduction to new areas is regularly reported, particularly in port cities, coastal areas, and islands (*6*). This expansion might explain the presence of *M. musculus domesticus* LCMV strains in Osaka and Jinxin, both coastal areas.

LCMV can take a severe toll on human health, particularly in immunosuppressed persons. Cases of death after organ transplant have been reported involving strains from both lineages I and II (*3*,*18*,*46*). Takagi et al. (*41*) showed that 3 LCMV strains—OQ28, WE, and BRC—differ in pathogenicity in mice, concluding that strains OQ28 and BRC were genetically classified within the same cluster but exhibited very different pathogenicity. In this study, we demonstrate that the OQ28 strain clusters to *M. musculus domesticus* lineage I and the BRC strain clusters to *M. musculus musculus* lineage II; thus, we propose the 2 lineages have different host origins. From this perspective, the differences observed in strain pathogenicity by Takagi et al. (*41*) seem less surprising. Nevertheless, the variation of pathogenicity of LCMV strains corresponding to other host taxa is currently unknown.

In conclusion, our results suggest that the evolutionary diversity of LCMV might reflect rodent expansion history. When a human LCMV infection is diagnosed, sampling efforts should be applied to any synanthropic rodents. This effort could help clarify LCMV evolutionary history and elucidate whether different lineages differ in their spillover ability.

Appendix 1 Additional data use in study of geographic distribution and evolution of lymphocytic choriomeningitis virus, central Europe.

Appendix 2 Additional information about geographic distribution and evolution of lymphocytic choriomeningitis virus, central Europe
