# Supplementary material for: New Perspective on the Geographic Distribution and Evolution of Lymphocytic Choriomeningitis Virus, Central Europe
Source: Emerg Infect Dis. 2021 Oct;27(10):2638–47. doi: 10.3201/eid2710.210224 (PMC8462312; doi:10.3201/eid2710.210224)
Supplement: Appendix 2 — Additional information about geographic distribution and evolution of lymphocytic choriomeningitis virus, central Europe [file 21-0224-Techapp-s2.pdf]

Article DOI: https://doi.org/10.3201/eid2710.210224

New Perspective on the Geographic Distribution and Evolution of Lymphocytic Choriomeningitis Virus, Central Europe

Appendix 2

Appendix 2 Table. Primers used in study of geographic distribution and evolution of lymphocytic choriomeningitis virus, central Europe

Identification of primers	Region of screening and length of fragment	Primer sequence	Reference
**LVL_3359D_Y+**	Large segment, 395 bp	5′- AGA ATC AGT GAA AGG GAA AGC AAY TC-3′	(*1*)
LVL_3359G_Y+		5′- AGA ATT AGT GAA AGG GAG AGT AAY TC-3′	
**LVL_3754A_R-**		5′- CAC ATC ATT GGT CCC CAT TTA CTA TGR TC-3′	
LVL_3754D_R-		5′- CAC ATC ATT GGT CCC CAT TTA CTG TGR TC-3′	
**OWS2805+**	Nucleoprotein, 648 bp	5′- GTC AGG CTT GGC ATT GTC CCA AAC TGR TTR TT-3′	(*2*)
OWS2810+		5′- CTT GGC ATT GTC CCA AAC TGR TTR TT-3′	
**OWS3400-**		5′- GCG CAC AGT GGA TCC TAG GC-3′	
OWS3400A-		5′- CGC AGA GTG GAT CCT AGG CTA TTK GAT TGC GC-3′	
LCMV_L_F1	Large segment, 442 bp	5′- TGR TCA TCA CTW GAH GTR TA-3′ (first round)	Nested assay, present study
LCMV-L_R1		5′- TRA GRA CHA ARA TGT TYA C-3′ (first round)	
**LCMV-L_F2**		5′- ATY CCY TGH CCC ATR TCA AT-3′ (second round)	
**LCMV-L_R2**		5′- TRG ARG AYT AYT TTG ARTC-3′ (second round)	
**OWS0001+**	Glycoprotein, 993 bp	5′-GCG CAC CGG GGA TCC TAG GC-3′	(*3*)
**OWS1000-**		5′-AGC ATG TCA CAA AAY TCY TCA TCA TG-3′	
**LCMV_IGR_L_F1**	Intergenic region, 408 bp	5′-GTC ATG CTG GCA GAG GTT TG-3′	Present study
**LCMV_IGR_L_R1**		5′-CTG TGA TGA CTG GCT GAG G-3′	

*Sequencing primers are indicated in bold. bp, base pair; +, forward primers; -, reverse primers.

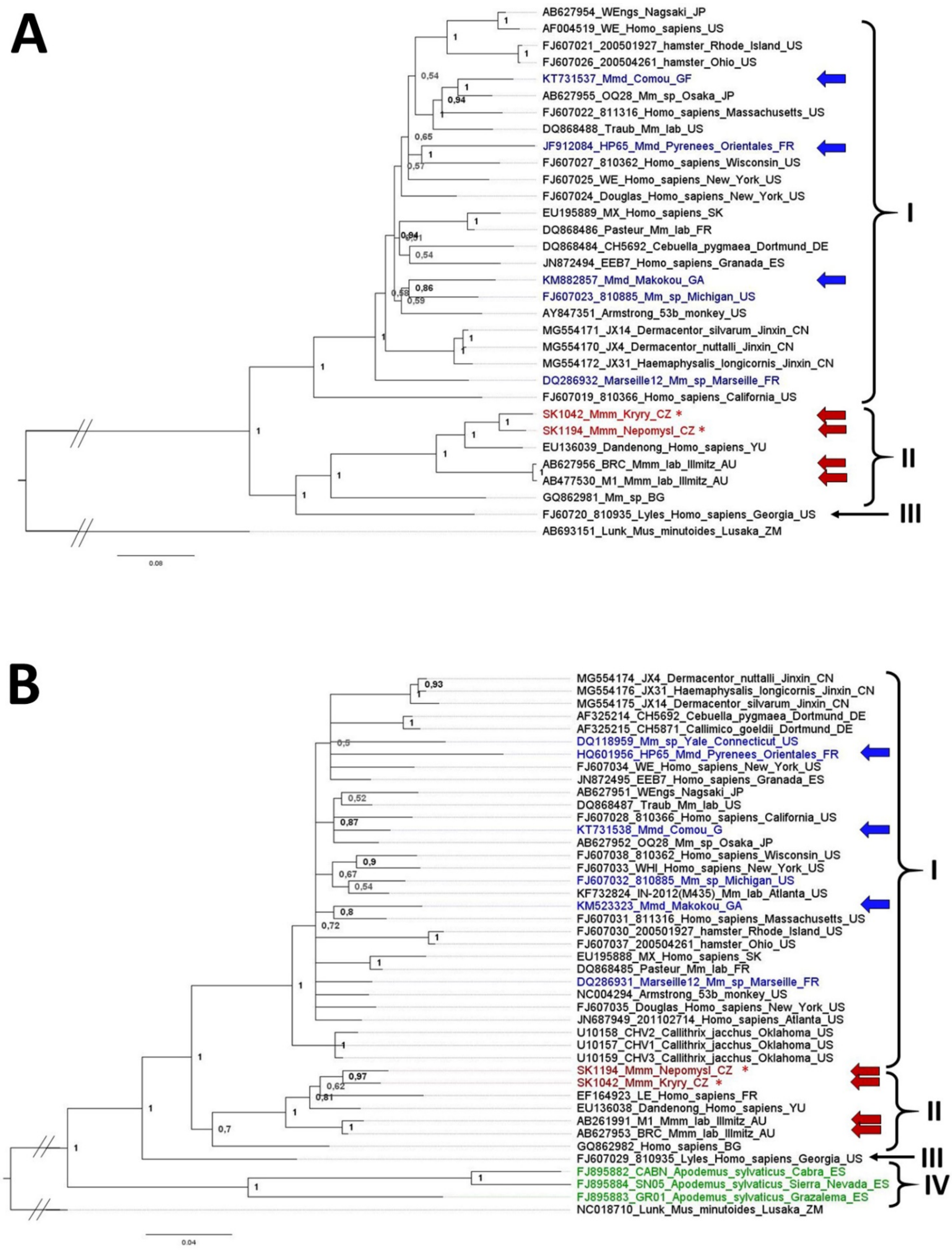
A
AB627954_WEngs_Nagsaki_JP
AF004519_WE_Homo_sapiens_US
FJ607021_200501927_hamster_Rhode_Island_US
FJ607026_200504261_hamster_Ohio_US
KT731537_Mmd_Comou_GF
AB627955_OQ28_Mm_sp_Osaka_JP
FJ607022_811316_Homo_sapiens_Massachusetts_US
DQ868488_Traub_Mm_lab_US
JF912084_HP65_Mmd_Pyrenees_Orientales_FR
FJ607027_810362_Homo_sapiens_Wisconsin_US
FJ607025_WE_Homo_sapiens_New_York_US
FJ607024_Douglas_Homo_sapiens_New_York_US
EU195889_MX_Homo_sapiens_SK
DQ868486_Pasteur_Mm_lab_FR
DQ868484_CH5692_Cebuella_pygmaea_Dortmund_DE
JN872494_EEB7_Homo_sapiens_Granada_ES
KM882857_Mmd_Makokou_GA
FJ607023_810885_Mm_sp_Michigan_US
AY847351_Armstrong_53b_monkey_US
MG554171_JX14_Dermacentor_silvarum_Jinxin_CN
MG554170_JX4_Dermacentor_nuttalli_Jinxin_CN
MG554172_JX31_Haemaphysalis_longicornis_Jinxin_CN
DQ286932_Marseille12_Mm_sp_Marseille_FR
FJ607019_810366_Homo_sapiens_California_US
SK1042_Mmm_Kryry_CZ *
SK1194_Mmm_Nepomysl_CZ *
EU136039_Dandenong_Homo_sapiens_YU
AB627956_BRC_Mmm_lab_Illmitz_AU
AB477530_M1_Mmm_lab_Illmitz_AU
GQ862981_Mm_sp_BG
FJ60720_810935_Lyles_Homo_sapiens_Georgia_US
AB693151_Lunk_Mus_minutoides_Lusaka_ZM
I
II
III
0.08
B
MG554174_JX4_Dermacentor_nuttalli_Jinxin_CN
MG554176_JX31_Haemaphysalis_longicornis_Jinxin_CN
MG554175_JX14_Dermacentor_silvarum_Jinxin_CN
AF325214_CH5692_Cebuella_pygmaea_Dortmund_DE
AF325215_CH5871_Callimico_goeldii_Dortmund_DE
DQ118959_Mm_sp_Yale_Connecticut_US
HQ601956_HP65_Mmd_Pyrenees_Orientales_FR
FJ607034_WE_Homo_sapiens_New_York_US
JN872495_EEB7_Homo_sapiens_Granada_ES
AB627951_WEngs_Nagsaki_JP
DQ868487_Traub_Mm_lab_US
FJ607028_810366_Homo_sapiens_California_US
KT731538_Mmd_Comou_G
AB627952_OQ28_Mm_sp_Osaka_JP
FJ607038_810362_Homo_sapiens_Wisconsin_US
FJ607033_WHI_Homo_sapiens_New_York_US
FJ607032_810885_Mm_sp_Michigan_US
KF732824_IN-2012(M435)_Mm_lab_Atlanta_US
KM523323_Mmd_Makokou_GA
FJ607031_811316_Homo_sapiens_Massachusetts_US
FJ607030_200501927_hamster_Rhode_Island_US
FJ607037_200504261_hamster_Ohio_US
EU195888_MX_Homo_sapiens_SK
DQ868485_Pasteur_Mm_lab_FR
DQ286931_Marseille12_Mm_sp_Marseille_FR
NC004294_Armstrong_53b_monkey_US
FJ607035_Douglas_Homo_sapiens_New_York_US
JN687949_201102714_Homo_sapiens_Atlanta_US
U10158_CHV2_Callithrix_jacchus_Oklahoma_US
U10157_CHV1_Callithrix_jacchus_Oklahoma_US
U10159_CHV3_Callithrix_jacchus_Oklahoma_US
SK1194_Mmm_Nepomysl_CZ *
SK1042_Mmm_Kryry_CZ *
EF164923_LE_Homo_sapiens_FR
EU136038_Dandenong_Homo_sapiens_YU
AB261991_M1_Mmm_lab_Illmitz_AU
AB627953_BRC_Mmm_lab_Illmitz_AU
GQ862982_Homo_sapiens_BG
FJ607029_810935_Lyles_Homo_sapiens_Georgia_US
FJ895882_CABN_Apodemus_sylvaticus_Cabra_ES
FJ895884_SN05_Apodemus_sylvaticus_Sierra_Nevada_ES
FJ895883_GR01_Apodemus_sylvaticus_Grazalema_ES
NC018710_Lunk_Mus_minutoides_Lusaka_ZM
I
II
III
IV
0.04

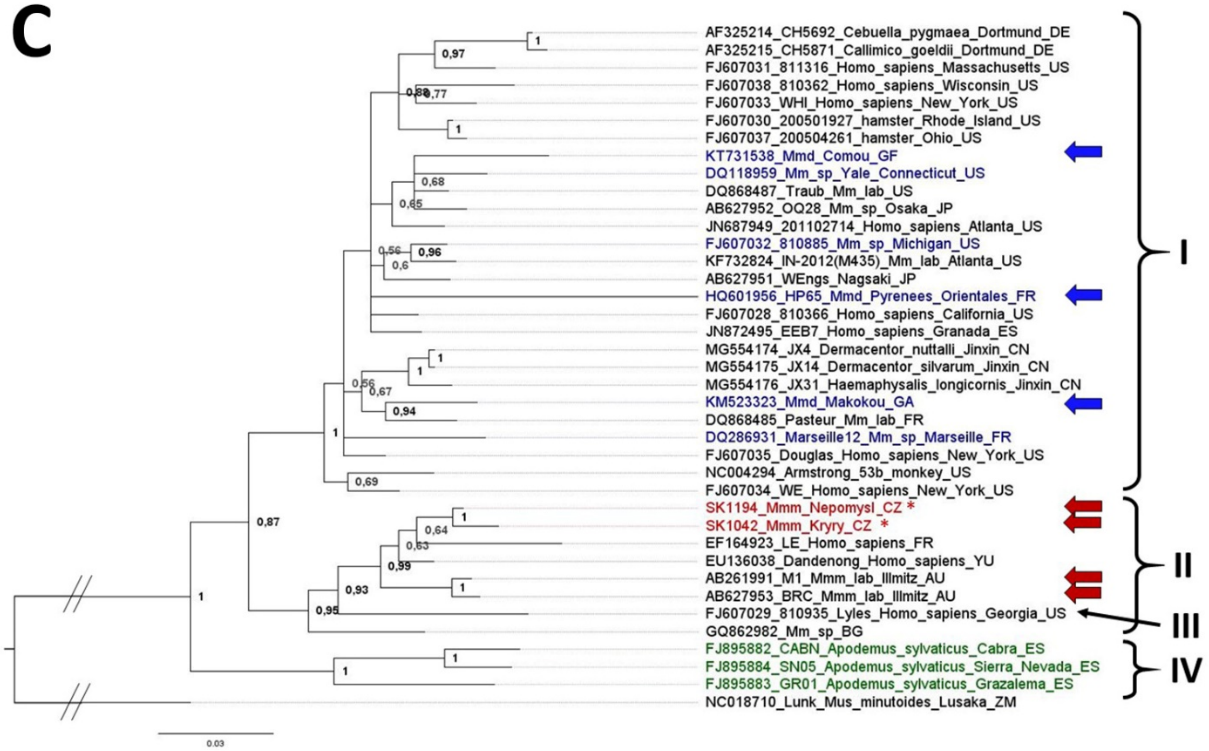

Appendix 2 Figure. Phylogenetic analyses performed on the amino acid sequences of lymphocytic choriomeningitis virus (LCMV) strains using Bayesian inference. Bayesian posterior probabilities were used to assess node support. Lunk virus from *Mus minutoides* (Africa) was used as outgroup. Names of LCMV strains are composed of GenBank number, strain name, host species, place and country of origin (if known), or isolation. Country code is defined as ISO code (https://countrycode.org). Colors indicate LCMV strains isolated from wild rodents where there is a match between expected mouse subspecies on the basis of geographic region and sampling area; blue indicates *Mus musculus domesticus*, red indicates *M. musculus musculus*. Arrow indicates known origin of mice subspecies on the basis of genetic data, asterix indicates LCMV strains from this study, and lineages are indicated by roman numerals. LCMV strains isolated from *Apodemus sylvaticus* are indicated in green (lineage IV). Scale bars indicate nucleotide substitutions per site. Mmd, *M. musculus domesticus*; Mmm, *M. musculus musculus*; Mmm_lab, laboratory mouse strain derived from *M. musculus musculus*; Mm_lab, laboratory mouse strain; Mm_sp, *Mus musculus* spp. A) Phylogenetic tree based on amino acid sequences of large gene, B) phylogenetic tree based on amino acid sequences of glycoprotein gene, C) phylogenetic tree based on amino acid sequences of nucleoprotein gene.

References

1. Vieth S, Drosten C, Lenz O, Vincent M, Omilabu S, Hass M, et al. RT-PCR assay for detection of Lassa virus and related Old World arenaviruses targeting the L gene. Trans R Soc Trop Med Hyg. 2007;101:1253–64. PubMed https://doi.org/10.1016/j.trstmh.2005.03.018

2. Coulibaly-N'Golo D, Allali B, Kouassi SK, Fichet-Calvet E, Becker-Ziaja B, Rieger T, et al. Novel arenavirus sequences in Hylomyscus sp. and Mus (Nannomys) setulosus from Côte d'Ivoire: implications for evolution of arenaviruses in Africa. PLoS One. 2011;6:e20893. PubMed https://doi.org/10.1371/journal.pone.0020893

3. Olschläger S, Lelke M, Emmerich P, Panning M, Drosten C, Hass M, et al. Improved detection of Lassa virus by reverse transcription-PCR targeting the 5′ region of S RNA. J Clin Microbiol. 2010;48:2009–13. PubMed https://doi.org/10.1128/JCM.02351-09
